# Characterization of *Brevibacillus laterosporus* Cas9 (BlatCas9) for Mammalian Genome Editing

**DOI:** 10.3389/fcell.2020.583164

**Published:** 2020-10-19

**Authors:** Ning Gao, Chengdong Zhang, Ziying Hu, Miaomiao Li, Jingjing Wei, Yongming Wang, Huihui Liu

**Affiliations:** ^1^Experimental Center of Forestry in North China, Chinese Academy of Forestry, Beijing, China; ^2^State Key Laboratory of Genetic Engineering, School of Life Sciences, Zhongshan Hospital, Obstetrics and Gynecology Hospital, Fudan University, Shanghai, China; ^3^Shanghai Engineering Research Center of Industrial Microorganisms, Shanghai, China

**Keywords:** CRISPR/Cas9, genome editing, compact, aav, gene therapy

## Abstract

Compact CRISPR/Cas9 systems that can be delivered by AAV for *in vivo* genome editing hold great promise for clinical applications. *Brevibacillus laterosporus* Cas9 (BlatCas9) is a compact Cas9 nuclease that has been identified for plant genome editing. Here, we characterize BlatCas9 as an alternative tool for mammalian genome editing. We demonstrate that BlatCas9 prefers a N4CNAA protospacer adjacent motif (PAM), but N4C PAM is also editable in mammalian cells. We next demonstrate that BlatCas9 enables genome editing in a variety of cell types. Furthermore, BlatCas9 can be packaged into AAV for genome editing. Finally, we characterize the specificity of BlatCas9. In summary, BlatCas9 offers an alternative tool for both basic research and clinical applications.

## Introduction

The CRISPR/Cas9 system is a versatile tool for genome editing, and it has been rapidly and widely adopted by the scientific community (Cong et al., 2013; Hwang et al., 2013; Mali et al., 2013; Xie et al., 2017; Wang et al., 2019). This is a two-component system that contains a Cas9 nuclease and a guide RNA (gRNA) (Jinek et al., 2012). They form a Cas9–gRNA complex, recognizing a gRNA complementary DNA sequence and generating a site-specific double-strand break (DSB) (Jinek et al., 2012; Cong et al., 2013; Mali et al., 2013). The DSB is repaired by the cell’s endogenous DNA repair machinery through either non-homologous end-joining (NHEJ) or homology-directed repair (HDR), resulting in site-specific mutations (Ran et al., 2013; Komor et al., 2017; Adli, 2018). By altering the 20-bp sequence at the 5′ end of gRNA, one can easily modify a new target in the genome. However, target site recognition also requires a specific protospacer adjacent motif (PAM) (Jinek et al., 2012; Karvelis et al., 2015; Leenay et al., 2016), which limits the targeting scope of Cas9 for precise positioning. In the last few years, a number of CRISPR/Cas proteins have been repurposed for genome editing (Adli, 2018; Teng et al., 2018, 2019; Liu et al., 2019; Tian et al., 2020). These CRISPR/Cas systems recognize different PAMs, expanding the targeting scope.

The majority of Cas9 variants are large proteins, which adds particular limitation when they are required to be packaged into adeno-associated viruses (AAVs) for *in vivo* gene therapy. For example, the most extensively applied SpCas9 protein is 1,366 aa, which, together with its gRNA sequence, are too large to be packaged into AAV (Wu et al., 2010). To overcome the size limitation, five smaller Cas9 variants (<1,100 aa) have been discovered for mammalian genome editing, including *Neisseria meningitides* Cas9 (NmCas9, 1,082 aa) (Esvelt et al., 2013; Hou et al., 2013), *Staphylococcus aureus* Cas9 (SaCas9, 1,053 aa) (Ran et al., 2015), *Campylobacter jejuni* Cas9 (CjCas9, 984 aa) (Kim et al., 2017), *Neisseria meningitidis* Cas9 (Nme2Cas9, 1,082 aa) (Edraki et al., 2019), and *Staphylococcus auricularis* (SauriCas9, 1,061 aa) (Hu et al., 2020). However, only a portion of genome sites can be targeted by these smaller Cas9 variants due to the PAM limitation.

Exploration of Cas9 orthologs could offer a diversity of PAM sequences and novel biochemical properties that may be beneficial for genome editing applications. *Brevibacillus laterosporus* Cas9 (BlatCas9, 1,092 aa) is a compact Cas9 nuclease that has been identified for plant genome editing (Karvelis et al., 2015). Interestingly, BlatCas9 recognizes N4CNDD PAM, which is different from the existing Cas9. In this study, we characterized BlatCas9 for mammalian genome editing. We characterized PAM preference and gRNA length in mammalian cells. We demonstrated that BlatCas9 enabled genome editing in a variety of mammalian cell types.

## Results

### BlatCas9 Enables Genome Editing in Mammalian Cells

**FIGURE 1:**
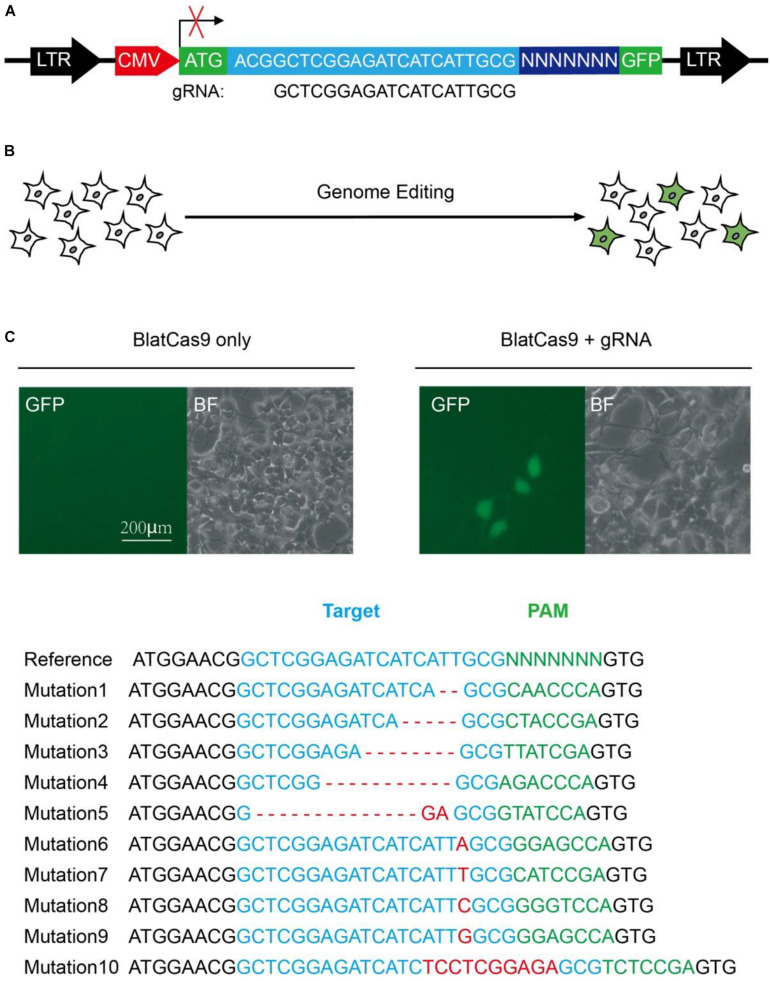
A GFP reporter assay for protospacer adjacent motif (PAM) screening. **(A)** Schematic diagram of the GFP reporter assay. A lentiviral vector containing a CMV-driven GFP is disrupted by the insertion of a target sequence followed by a 7-bp random sequence between ATG and GFP coding sequence. The library DNA is stably integrated into the HEK293T cells. Genome editing generates in-frame mutations for a portion of cells, leading to GFP expression. **(B)** Transfection of *Brevibacillus laterosporus* Cas9 (BlatCas9) with guide RNA (gRNA) results in GFP expression, while transfection of BlatCas9 alone does not induce GFP expression. **(C)** Deep sequencing shows that the target sequences with various PAM sequences can be edited. The target sequence is shown in blue; indel mutations are shown in red; 7-bp random sequences are shown in green.

To test whether BlatCas9 enables genome editing in mammalian cells, we employed a GFP-activation approach that allowed testing Cas9 activity in mammalian cells (Figure 1A) (Hu et al., 2020). We synthesized the gRNA scaffold and human-codon-optimized BlatCas9, flanked by nuclear localization signal sequences (NLS), and transfected them into the reporter cells (Supplementary Figures S1, S2). When we transfected BlatCas9 alone, no GFP-positive cells were observed; when we transfected BlatCas9 together with a 20-bp gRNA, GFP-positive cells were observed (Figure 1B). GFP-positive cells were sorted out, and sequences containing 7-bp randomized DNA were PCR-amplified for deep sequencing. Sequencing results revealed that insertions/deletions (indels) occurred (Figure 1C), demonstrating that BlatCas9 enables genome editing in mammalian cells.

### Identification of BlatCas9 PAM Preference in Mammalian Cells

**FIGURE 2:**
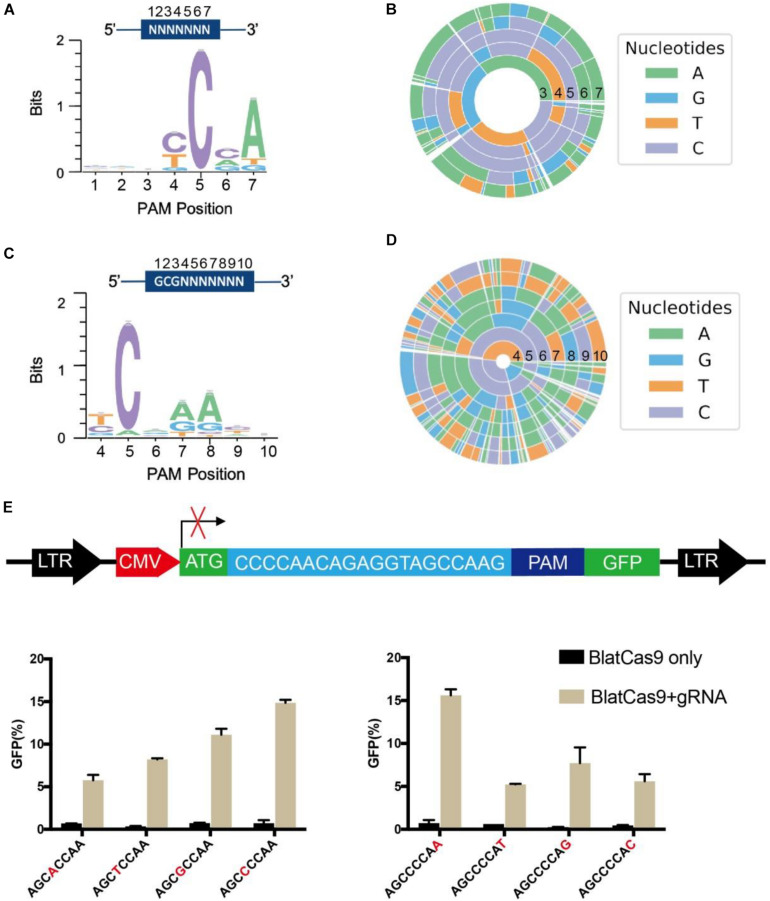
PAM sequence analysis for BlatCas9. **(A,B)** WebLogo and PAM wheel are generated from the first round of PAM screening. **(C,D)** WebLogo and PAM wheel are generated from the second round of PAM screening. **(E)** Schematic diagram of the GFP reporter assay for PAM tests. BlatCas9 can accept any nucleotide at positions 4 and 8 of PAM. PAM sequences are shown below the figures; nucleotides at positions 4 and 8 are shown in red; data are shown as mean ± SD. *n* = 3.

Protospacer adjacent motif sequences play a crucial role in target recognition. To characterize BlatCas9 PAM sequences in mammalian cells, we generated interactive visualization schemes (a WebLogo and a PAM wheel) based on deep-sequencing data (Leenay et al., 2016). Both of them revealed that BlatCas9 strongly preferred C at position 5, A at position 7, and accepted any nucleotide at positions 1–3 of PAM (Figures 2A,B). To test whether BlatCas9 requires longer PAM, we shifted the target sequence by three nucleotides in the 5′ direction to allow PAM identification to be extended from 7 to 10 bp. The results revealed that BlatCas9 preferred N4CNAA PAM, where C was strongly preferred at position 5, and A was mildly preferred at positions 7 and 8 (Figures 2C,D). These results are very similar to an *in vitro* PAM library screening results, which reveal that BlatCas9 prefers NNNNCNDD (N = G, C, A, or T; D = A, G, or T) PAM (Karvelis et al., 2015). We observed that A was preferred stronger in the first round of screening than that in the second round. In the first round of screening, a G was fixed at position 8, which may influence the nucleotide preference at position 7. BlatCas9 had no significant nucleotide preference at PAM longer than eight nucleotides.

To further test the PAM preference of BlatCas9, we constructed another PAM library with 8 bp of randomized DNA sequences (Supplementary Figure S3A). We fixed the first 3 bp of the PAM sequence as CTG and tested the PAM preference for other positions. The deep sequencing result revealed that BlatCas9 preferred N4CNAA PAM (Supplementary Figures S3B,C).

To test whether BlatCas9 can accept any nucleotide at positions 4 and 8 of PAM, we inserted a protospacer sequence with varied nucleotides at positions 4 and 8 of PAMs into GFP reporter plasmids and established stable cell lines (Figure 2E). Transfection of BlatCas9 with the corresponding gRNA induced GFP expression for all of them (Figure 2E), indicating that BlatCas9 can accept any nucleotide at these two positions. To test whether BlatCas9 can accept C at position 7 of PAM, we generated three PAMs with C at position 7. Transfection of BlatCas9 with the corresponding gRNA induced GFP expression for all of them (Supplementary Figure S4), indicating that BlatCas9 can accept C at position 7. In conclusion, N4C PAM is also editable by BlatCas9.

### BlatCas9 Enables Editing Endogenous Genomic Sites

**FIGURE 3:**
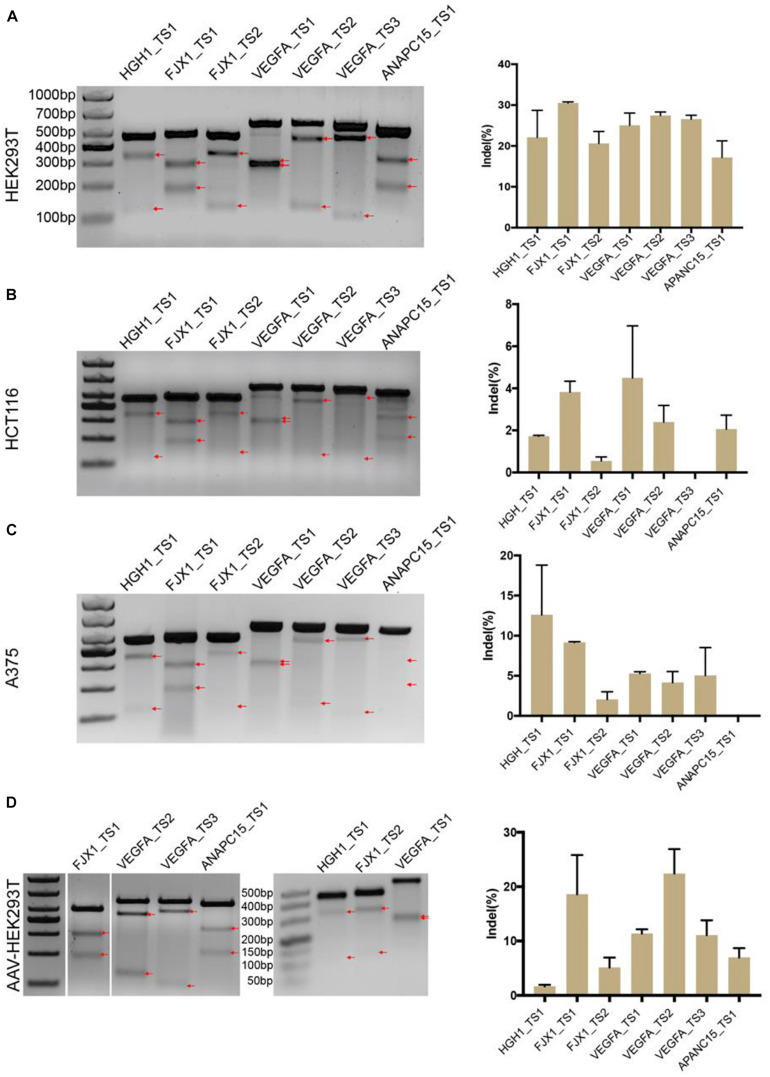
Genome-editing capability of BlatCas9. BlatCas9 enables genome editing for a list of seven endogenous target sites in **(A)** HEK293T, **(B)** HCT116, and **(C)** A375 cells, respectively. **(D)** BlatCas9 enables genome editing for a list of seven endogenous target sites by adeno-associated virus (AAV) delivery in HEK293T cells. Gel pictures of T7E1 digestion are shown on the left; the bands generated by T7EI digestion are indicated by red arrows. Indel frequencies measured by targeted deep sequencing are shown on the right. Data are shown as mean ± SD. *n* = 3.

We next tested the genome-editing capability of BlatCas9 with a panel of seven endogenous gene targets in three cell lines, HEK293T, HCT116, and A375. The results showed that BlatCas9 could generate indels at all seven endogenous loci in HKE293T cells (Figure 3A) and varied indel efficiencies in HCT116 and A375 cells (Figures 3B,C). Importantly, BlatCas9 could be packaged into AAV for efficient genome editing in HEK293T cells (Figure 3D). In addition, we compared the efficiency of BlatCas9 with SpCas9 at three loci. BlatCas9 showed lower efficiency at ANAPC15_TS2, higher efficiency at ANAPC15_TS3, and similar efficiency at ANAPC15_TS4 compared to SpCas9 (Supplementary Figure S5). Taken together, BlatCas9 offers a novel platform for genome editing.

### BlatCas9 Promotes Homologous Recombination

**FIGURE 4:**
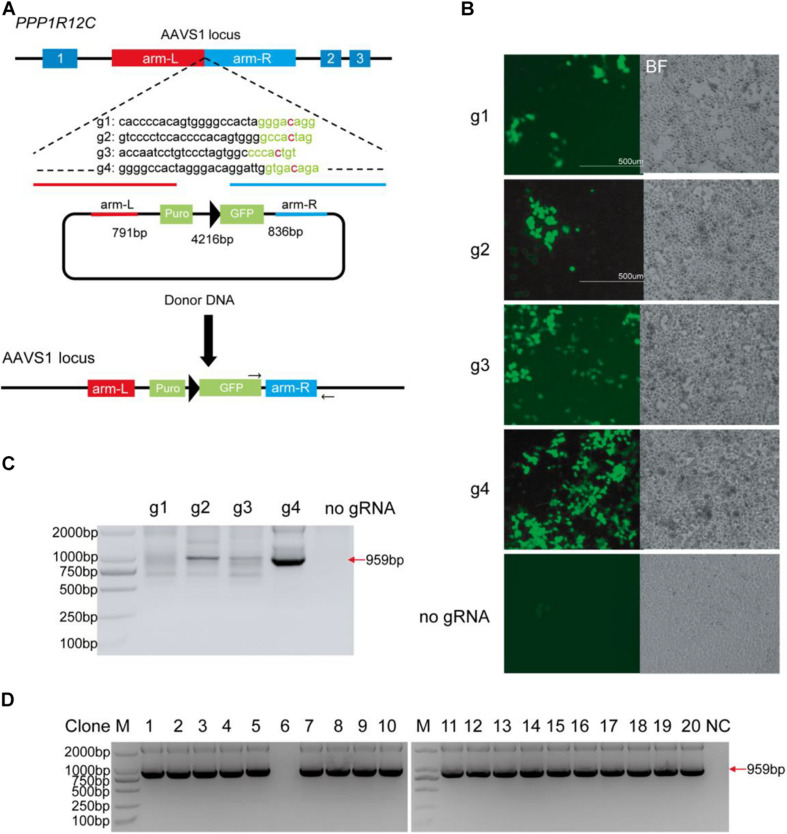
BlatCas9 promotes homologous recombination. **(A)** A schematic diagram of homologous recombination using BlatCas9. **(B)** Thirty days after transfection, GFP-positive cells can be observed. Cells transfected with donor plasmid alone are used as control. Notably, control cells are not selected with drugs, and only a few GFP-positive cells can be observed. **(C)** Targeted integration in mixed cells is confirmed by polymerase chain reaction (PCR). **(D)** Targeted integration in the single cell-derived clones is confirmed by PCR. M, DNA marker; NC, negative control where genomic DNA was unedited.

To test whether BlatCas9 can promote homologous recombination, we designed four gRNAs targeting the AAVS1 locus. We employed a donor plasmid containing a GFP reporter and a promoterless puromycin cassette, which expresses the puromycin resistance element only if inserted downstream of the PPP1R12C promoter (AAVS1 locus) (Figure 4A) (Wang et al., 2012; Chen et al., 2016). The donor plasmid with individual gRNA was transfected into cells followed by drug selection. Thirty days after tranfection, stable GFP-expressing cell lines were established (Figure 4B). Very few GFP-positive cells could be observed for cells transfected with donor plasmid alone due to the random integration. To confirm that targeted integration occurred, genomic DNA was extracted for polymerase chain reaction (PCR) detection. One primer targeted the GFP gene, and the other primer targeted the genomic DNA. If targeted integrations occur, a 959-bp band will be present. The results revealed that gRNA #4 (g4) induced efficient targeted integration (Figure 4C). For cells edited by g4, we further tested targeted integration for single cell-derived clones. Of 20 clones, 19 clones contained targeted integration (Figure 4D).

### Optimization of BlatCas9 Guide Length

**FIGURE 5:**
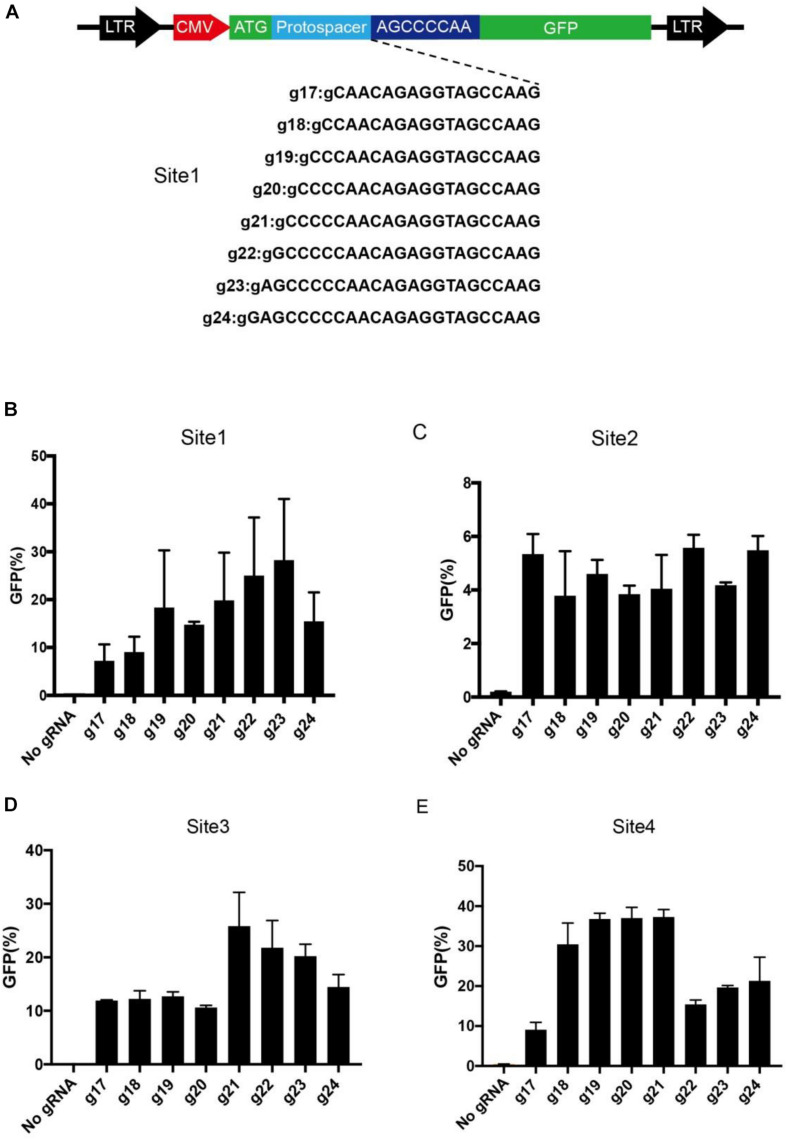
Optimization of gRNA length. **(A)** A target sequence is inserted into the GFP reporter construct. A list of eight gRNAs varying from 17 to 24 bp is tested for genome editing. Additional G is added for transcription by a U6 promoter. **(B–E)** Quantification of GFP-positive cells for four different target sites. Data are shown as mean ± SD. *n* = 3.

We next optimized the guide length of BlatCas9 for genome editing. We used a GFP-activation system to measure editing efficiency. A target sequence (site 1) was inserted into the GFP reporter to induce frameshift mutation and established a stable cell line (Figure 5A). When editing occurred, in-frame mutation can occur, leading to GFP expression. We cotransfected BlatCas9 together with a series of gRNAs with variable guide lengths (17–24 bp) into cells and measured GFP-positive cells by fluorescence-activated cell sorting (FACS). The first nucleotide in the gRNAs was fixed to extra guanine (G) so that gRNAs can be transcribed by the U6 promoter. The results revealed that BlatCas9 was active for all gRNAs, but the 23-bp guide was the most active (28.3% GFP-positive cells) (Figure 5B). We tested three additional targets. However, the optimal guide length depends on the target sequences. For site 2, all gRNAs displayed similar efficiency. For site 3, the optimal guide length was 21 bp. For site 4, the optimal guide length was 18–21 bp (Figures 5C–E).

### Specificity Analysis of BlatCas9

**FIGURE 6:**
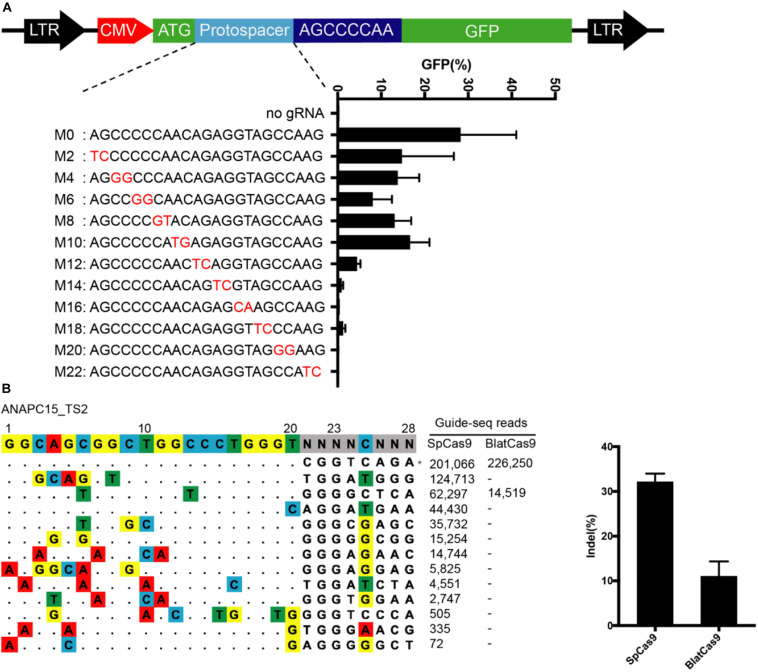
Analysis of BlatCas9 specificity. **(A)** A target sequence is inserted between the ATG and GFP coding sequence, disrupting GFP expression. GFP expression can be induced by genome editing. A panel of gRNAs with dinucleotide mismatches (red), and each gRNA activity is shown below. *n* = 2. **(B)** GUIDE-seq is performed to compare the off-target effects of SpCas9 and BlatCas9. A target site (targeting *ANAPC15*) with a PAM compatible for both nucleases is selected. Read numbers for on and off targets are shown on the right. Mismatches compared to the on-target site are shown and highlighted in color. On-target site is indicated by “*”.

We next evaluated the off-target activity of BlatCas9 by using the GFP-activation cell line (Figure 6A). We initially generated a panel of 20-bp guides with single nucleotide mutation. BlatCas9 could tolerate single nucleotide mismatch at positions 1–18 but not position 20, counting the PAM as positions 21–28 (Supplementary Figure S6). We next generated a panel of 23-bp guides with dinucleotide mutations (Figure 6A). BlatCas9 showed reduced activity with mismatches at positions 1–11 and showed minimal or no activity with mismatches at positions 13–22, counting the PAM as positions 24–31.

To compare the genome-wide off-target effects of BlatCas9 to that of SpCas9, the genome-wide, unbiased identification of DSBs enabled by sequencing (GUIDE-seq) was performed (Tsai et al., 2015). We selected a target site containing a PAM that can be recognized by both SpCas9 and BlatCas9 on *ANAPC15*. Following transfections of Cas9 + gRNA plasmids and GUIDE-seq oligos, we prepared libraries for deep sequencing. Sequencing and analysis revealed that on-target cleavage occurred for both Cas9 orthologs, reflected by GUIDE-seq read counts (Figure 6B). We identified 12 off-target sites for SpCas9. In contrast, we only identified one off-target site for BlatCas9. BlatCas9 requires longer PAM, which may contribute to less off-target effects in mammalian cells.

## Discussion

Small Cas9 nucleases (<1,100 aa) can be delivered by AAV for *in vivo* genome editing and hold great promise for gene therapy. Although five small CRISPR/Cas9 systems have been employed for mammalian genome editing, the targeting scope remains limited due to the requirement for a PAM sequence (Jinek et al., 2012; Karvelis et al., 2015; Leenay et al., 2016). These five small CRISPR/Cas9 systems include SaCas9 (Ran et al., 2015), NmCas9 (Hou et al., 2013), CjCas9 (Kim et al., 2017), Nme2Cas9 (Edraki et al., 2018), and SauriCas9, which recognize target sequences associated with NNGRRT, N4GAYW/N4GYTT/N4GTCT, N4RYAC, N4CC, and NNGG PAMs, respectively. Exploration of Cas9 orthologs from different bacteria is another way to increase the targeting scope.

BlatCas9 is a compact Cas9 nuclease that has displayed activity *in vitro* and in plants (Karvelis et al., 2015). In this study, we demonstrate that BlatCas9 also enables genome editing in mammalian cells, extending the list of small CRISPR/Cas9 tools. Interestingly, our GFP reporter assay reveals that N4C PAM is editable by BlatCas9, expanding the targeting scope. We observed that BlatCas9 tolerates dinucleotide mismatches at positions 1–11, indicating that the specificity remains to be improved. Several strategies, including rational design and directed evolution, have been used for Cas9 specificity improvement (Kleinstiver et al., 2016; Slaymaker et al., 2016; Chen et al., 2017; Casini et al., 2018; Vakulskas et al., 2018). These strategies can also be used to improve BlatCas9 specificity in future work. With further development, we anticipate that BlatCas9 can be an important genome editing tool for both basic research and clinical applications.

## Materials and Methods

### Cell Culture and Transfection

A375 and HEK293T cells were maintained in Dulbecco’s modified Eagle medium (DMEM) supplemented with 10% FBS (Gibco), 100 U/ml penicillin, and 100 mg/ml streptomycin at 37°C and 5% CO_2_. HCT116 cells were maintained in McCoy’s 5A supplemented with 10% FBS (Gibco), 100 U/ml penicillin and 100 mg/ml streptomycin at 37°C and 5% CO_2_. For BlatCas9 PAM sequence screening, HEK293T cells were plated in 10-cm dishes and transfected at ∼60% confluency with BlatCas9-gRNA-expressing plasmid (15 μg) using Lipofectamine 2000 (Life Technologies). For genome editing of BlatCas9, cells were seeded on 12-well plates and transfected with BlatCas9-gRNA plasmid (1 μg) using Lipofectamine 3000 in Opti-MEM according to the manufacturer’s instructions. The transfected A375, HEK293T and HCT116 cells were selected using media supplemented with 10, 10, 10, and 7 μg/ml of blasticidin, respectively.

### Plasmid Construction

BlatCas9-gRNA expression plasmid construction: the vector backbone of pX601 (Addgene #107055) was used to express Cas9. First, the miniCMV promoter on pX601 was replaced by normal CMV promoter as follows: pX601 was digested with *Xba*I/Age1 to remove miniCMV promoter; normal CMV promoter was PCR-amplified from pCMV–ABEmax plasmid (Addgene #125648) using primers CMV-F/CMV-R, and cloned into linearized pX601 by T4 DNA ligation (NEB), resulting in the pAAV–CMV–SaCas9 plasmid. Second, the AAV–CMV–SaCas9 plasmid was PCR amplified by using primers pX601-F/pX601-R to remove SaCas9; human codon-optimized BlatCas9 gene was synthesized by HuaGene (Shanghai, China); BlatCas9 was cloned into the AAV–CMV–SaCas9 backbone by NEBuilder Assembly Tool (NEB) following the manufacturer’s instructions, resulting in AAV–CMV–BlatCas9. For the genome editing of BlatCas9, the fragment of the human codon-optimized BlatCas9 gene and the blasticidin gene was synthesized by HuaGene (Shanghai, China); the fragment was cloned into the AAV–CMV–SaCas9 backbone by the NEBuilder Assembly Tool (NEB) following the manufacturer’s instructions, resulting in AAV–CMV–BlatCas9–BSD. Sequences were verified by Sanger sequencing (GENEWIZ, Suzhou, China). The human-codon-optimized BlatCas9 sequence is available in Supplementary Figure S2. All target sequences can be found in Supplementary Table S1; all primers can be found in Supplementary Table S2.

### PAM Sequence Analysis

Twenty base-pair sequences flanking the target sequence were used to fix the target sequence. Three nucleotides in front of a random sequence and GTGAGCAAGGGCG AGGAGCT were used to fix the 7-bp random sequence. Target sequences with in-frame mutations were used for PAM analysis. The 7-bp random sequence was extracted and visualized by WebLog3 (Crooks et al., 2004) and PAM wheel chart to demonstrate PAMs (Leenay et al., 2016).

### Verification of PAM Sequence With GFP Reporter Constructs

Three GFP reporter plasmids containing different targets CTGGTCAGGAATGATCTGGAGACCCAGA, CCCCAACAG AGGTAGCCAAGAGCCCCAA and GGTCGAAGTTGGCCG TCAGGTGGTCGAA were constructed. Each plasmid was packed into a lentivirus to generate stable cell lines. To remove background mutations that induce GFP expression, the GFP-negative cells were sorted by the MoFlo XDP machine. The sorted cells were seeded into 24 wells and transfected with AAV–CMV–BlatCas9–BSD plasmid (800 ng) by Lipofectamine 2000 (Life Technologies). Three days after editing and selecting (10 μg/ml of blasticidin), the GFP-positive cells were analyzed on the Calibur instrument (BD). Data were analyzed using FlowJo.

### Genome Editing of BlatCas9 at Endogenous Sites in Different Cell Lines

A375, HEK293T, and HCT116 cells were seeded into 12 wells and transfected with AAV–CMV–BlatCas9–BSD (1 μg) by Lipofectamine 3000. The transfected A375, HEK293T, and HCT116 cells were selected using media supplemented with 10, 10, and 7 μg/ml of blasticidin, respectively. Cells were collected 3 days after transfection and selection. The genomic DNA was isolated, and the target sites were PCR amplified and extracted by Gel Extraction kit (QIAGEN). The PCR products were subjected to deep sequencing to check the editing efficiency.

### Test of BlatCas9 Specificity

To test the specificity of BlatCas9, we generated a GFP reporter cell line with AGCCCCAA PAM. The HEK293T cells were seeded into 12 wells and transfected with AAV–CMV–BlatCas9–BSD (1 μg) by Lipofectamine 2000 (Life Technologies). Three days after editing and selecting (10 μg/ml of blasticidin), the GFP-positive cells were analyzed on a Calibur instrument (BD). Data were analyzed using FlowJo.

### Adeno-Associated Virus Production

For the seven individual endogenous target site BlatCas9–gRNA packaging, HEK293T cells were seeded at ∼40% confluency in a 6-cm dish the day before transfection. For each well, 2 μg of expressing plasmid, 2 μg of pAAV-RC (GenBank: AF369963), and 4 μg of pAAV-helper were transfected using 80 μl of PEI (0.1% m/v, Polysciences, Cat# 23966, pH 4.5). The media was changed 8 h after transfection. After 72 h, cells were scraped and poured into a 15-ml conical centrifuge tube. They were spun at 3,000 rpm, at 4°C for 10 min, and the supernatant was transferred into a new 15-ml tube. The cell pellets were resuspended in 1 ml of RB TMS buffer (50 mM Tris-HCl, 150 mM NaCl, pH 8.0), then transferred to a new 15-ml conical tube. They were frozen in a dry ice-ethanol bath for 10 min and thawed at 37°C for 10 min and repeated three times. The cells were spun at 3,000 rpm, at 4°C for 10 min. The two supernatants were mixed and filtered with a 0.45-μm polyvinylidene fluoride filter. One half of the volume of the mixed solution (1M NaCl + 10% PEG8000) was added and incubated at 4°C overnight. After centrifugation at 4°C for 2 h at 12,000 rpm, the flow-through was discarded, and 200 μl of chilled RB TMS was added. The quantitative PCR reveals that AAV titration is 6.3 × 10^8^ copies/μl. Sixty microliters of the virus was added into a 12-well plate with ∼80% confluency of HEK293T.

### Guide-Seq

GUIDE-seq experiments were performed as described previously (Tsai et al., 2015), with minor modifications. Briefly, 2 × 10^5^ of HEK293T cells were transfected with 1 μg of AAV–CMV–BlatCas9–BSD plasmid and 100 pmol of annealed GUIDE-seq oligonucleotides by electroporation, and then the cells were seeded into a 12-well plate. Electroporation parameters (voltage, width, number of pulses) were 1,150 V, 30 ms, and 1 pulse. Genomic DNA was extracted with a DNeasy Blood and Tissue kit (QIAGEN) 5 days after transfection according to the manufacturer’s protocol. Library preparation and sequencing were performed exactly as described previously (Tsai et al., 2015).

### Quantification and Statistical Analysis

All the data are shown as mean ± SD. Statistical analyses were conducted using Microsoft Excel.

## Data Availability Statement

All datasets generated for this study are included in the article/Supplementary Material.

## Author Contributions

NG, ZH, ML, and JW performed the experiments. CZ analyzed the data. YW designed the experiments and wrote the manuscript. YW revised the manuscript. YW and HL supervised the project. HL applied for grants. All authors read and approved the final manuscript.

## Conflict of Interest

The authors declare that the research was conducted in the absence of any commercial or financial relationships that could be construed as a potential conflict of interest.
