# Supplementary material for: Characterization of Brevibacillus laterosporus Cas9 (BlatCas9) for Mammalian Genome Editing
Source: Front Cell Dev Biol. 2020 Oct 19;8:583164. doi: 10.3389/fcell.2020.583164 (PMC7604293; doi:10.3389/fcell.2020.583164)
Supplement: Supplementary file 1 [file Data_Sheet_1.PDF]

Supplemental Information

Characterization of Brevibacillus Laterosporus Cas9 (BlatCas9) for Mammalian Genome Editing

Ning Gao, Chengdong Zhang, Ziying Hu, Miaomiao Li, Jingjing Wei, Yongming Wang, Huihui Liu

GAGGGCCTATTTCCCATGATTCCTTCATATTTGCATATACGATACAAGGCTGT
TAGAGAGATAATTGGAATTAATTTGACTGTAAACACAAAGATATTAGTACAA
AATACGTGACGTAGAAAGTAATAATTTCTTGGGTAGTTTGCAGTTTTAAAAT
TATGTTTTAAAATGGACTATCATATGCTTACCGTAACTTGAAAGTATTTCGAT
TTCTTGGCTTTATATATCTTGTGGAAAGGACGAAACACCGCACGACGGTAA
ACACCCTCGCTATAGTTCCTTACTGAAAGGTAAGTTGCTATAGTAAGGGCA
ACAGACCCGAGGCGTTGGGGATCGCCTAGCCCGTGTTTACGGCTCTCCCC
ATATTCAAAATAATGACAGACGAGCACCTTGGAGCATTTATCTCCGAGGTG
CTTTTTTT

Figure S1. BlatCas9 gRNA expression cassette, Related to Figure 1.
Human U6 polymerase III promoter region is shown in black font. The region encoding the spacer is blue highlighted black font. The repeat, self-folding tetraloop, anti-repeat and 3’ tracrRNA region are indicated in green highlighted black font. The human U6 polymerase III terminator is represented by red font.

ATGGCCCCAAAGAAGAAGCGGAAGGTCGGTATCCACGGAGTCCCAGCAGC
CTACACCATGGGAATCGACGTGGGCATTGCCAGCTGTGGCTGGGCCATCGT
GGATTTGGAGCGCCAGAGAATTATAGATATAGGCGTGCGGACTTTTGAAAA
AGCTGAAAATCCAAAGAACGGAGAGGCCCTGGCAGTGCCCCGGCGGGAG
GCCAGGTCCAGCAGGCGCCGCCTGCGGCGCAAGAAACACCGCATTGAGA
GGCTGAAGCACATGTTTGTGAGGAATGGCCTGGCTGTGGACATCCAGCATC
TGGAACAAACCCTCAGGAGCCAGAATGAAATTGATGTGTGGCAGCTGCGC
GTGGATGGATTGGACAGGATGCTGACTCAGAAGGAGTGGCTGAGGGTCCT
GATACATTTGGCTCAGAGGCGGGGCTTCCAGAGCAATAGGAAGACCGATG
GTTCTTCTGAGGATGGGCAGGTGCTCGTCAATGTCACTGAGAATGACAGAC
TGATGGAAGAGAAAGACTACAGAACTGTGGCAGAAATGATGGTGAAGGAT
GAAAAGTTTTCTGACCACAAGAGAAATAAAAATGGGAACTACCACGGCGT
CGTGTCTAGAAGCTCCCTGCTGGTGGAGATTCACACCCTGTTTGAAACCCA
GCGGCAGCACCACAACAGCCTGGCCTCCAAGGACTTTGAGCTTGAATATG
TGAACATCTGGAGCGCCCAAAGGCCAGTGGCCACTAAGGATCAGATAGAA
AAGATGATTGGCACCTGCACCTTTCTGCCTAAGGAGAAGCGCGCCCCAAA
AGCATCCTGGCACTTCCAGTACTTCATGTTGCTGCAGACTATCAATCACATC
AGGATCACTAACGTCCAGGGGACCAGGTCTCTCAACAAGGAGGAAATAGA
GCAGGTGGTGAATATGGCTCTGACCAAGTCCAAAGTGAGCTACCATGATAC
TCGGAAAATTCTTGACCTCTCAGAAGAATACCAGTTTGTTGGGCTGGACTA
CGGGAAGGAAGATGAAAAGAAGAAAGTTGAAAGTAAAGAAACCATCATA
AAACTGGATGACTATCATAAACTCAATAAGATTTTTAATGAGGTGGAACTTG
CCAAAGGTGAGACCTGGGAGGCAGATGATTATGATACAGTGGCTTATGCTC
TCACCTTCTTTAAGGACGATGAGGACATCCGGGACTATCTCCAGAATAAATA
CAAAGACTCAAAGAATAGACTGGTCAAAAATTTGGCCAACAAGGAGTATA
CTAATGAACTCATTGGAAAGGTGTCCACACTGTCCTTCAGGAAGGTTGGCC
ACCTGTCTTTGAAGGCGCTGAGAAAGATCATCCCCTTCCTCGAACAGGGCA

TGACATACGACAAGGCCTGCCAGGCTGCTGGATTTGATTTTCAGGGAATTA
GCAAGAAGAAAAGATCTGTGGTTCTTCCCGTGATTGACCAGATCAGCAAC
CCTGTTGTCAACCGCGCACTGACGCAGACCCGGAAGGTCATCAACGCCCT
CATCAAGAAGTATGGAAGCCCTGAGACCATTCATATCGAGACTGCCAGAGA
GCTCAGCAAAACATTTGACGAAAGGAAAAACATTACAAAAGATTATAAAG
AAAACAGAGATAAGAACGAGCACGCAAAGAAGCATCTCTCTGAACTGGGC
ATCATTAACCCAACAGGCCTCGACATTGTCAAGTACAAGCTGTGGTGTGAG
CAGCAAGGCCGCTGCATGTATTCCAACCAGCCTATTTCCTTTGAACGACTG
AAAGAATCTGGATACACAGAAGTGGACCATATTATTCCCTACAGCCGCAGC
ATGAATGATTCCTATAACAACCGGGTGCTGGTGATGACCAGAGAAAATAGG
GAGAAAGGGAATCAGACACCATTTGAGTACATGGGCAATGACACACAGCG
CTGGTATGAGTTTGAGCAGCGAGTGACCACCAATCCCCAGATTAAAAAGG
AAAAAAGGCAGAACCTCCTGCTCAAGGGCTTTACCAACAGGCGAGAGCTG
GAGATGCTTGAGAGAAACCTGAACGACACCCGCTACATCACCAAATATTTG
AGCCACTTTATCTCCACCAACTTGGAATTTAGCCCCAGTGATAAAAAGAAG
AAGGTGGTAAACACCAGCGGCCGGATCACCTCTCACCTCCGCTCCAGGTG
GGGCCTAGAGAAGAACCGAGGCCAGAACGATCTTCATCATGCCATGGATG
CCATCGTCATTGCTGTCACCAGTGACAGTTTCATTCAGCAGGTTACAAATTA
CTATAAGCGAAAGGAAAGACGCGAGTTGAACGGGGACGACAAATTTCCTC
TGCCCTGGAAGTTCTTCCGGGAGGAGGTGATCGCCCGGCTGAGCCCAAAC
CCCAAGGAGCAAATTGAGGCTTTGCCCAATCATTTCTACAGTGAAGACGAG
CTGGCCGACCTTCAGCCCATCTTTGTCAGCAGAATGCCCAAACGGAGCATC
ACAGGAGAAGCCCACCAGGCCCAGTTCCGCAGGGTGGTGGGAAAAACCA
AGGAGGGCAAAAATATTACTGCAAAAAAGACAGCCCTGGTTGATATTTCTT
ATGACAAGAATGGAGACTTCAACATGTACGGCAGGGAAACAGACCCCGCC
ACCTATGAAGCTATCAAAGAGCGGTACCTGGAGTTCGGAGGAAATGTGAA
AAAAGCCTTCTCCACTGACCTGCACAAACCCAAGAAAGATGGCACTAAAG
GCCCCCTGATTAAGTCTGTCCGCATCATGGAGAACAAAACTTTGGTGCACC
CTGTGAACAAGGGCAAGGGGGTGGTCTACAACAGTTCCATTGTTAGAACA
GATGTGTTCCAAAGAAAAGAGAAGTACTACCTCCTCCCGGTGTACGTGACT
GATGTCACAAAGGGCAAGCTCCCCAACAAAGTAATTGTGGCCAAGAAAGG
CTACCACGACTGGATCGAAGTAGATGATAGCTTCACCTTCCTGTTCAGCCTC
TACCCAAATGATCTGATCTTCATCAGACAAAATCCTAAAAAAAAAATCTCTC
TGAAGAAGAGGATTGAATCCCACAGCATTTCAGATTCTAAAGAAGTTCAGG
AGATCCACGCCTATTACAAGGGAGTGGACAGCAGCACAGCTGCCATTGAAT
TCATCATCCATGACGGCAGCTATTATGCCAAGGGTGTTGGTGTGCAGAATCT
GGACTGCTTCGAGAAATATCAGGTCGATATCCTGGGAAACTATTTCAAGGT
AAAAGGGGAGAAGCGGCTCGAGCTGGAGACATCTGATTCAAACCACAAG
GGGAAAGATGTTAACTCCATCAAGAGCACCTCCAGATTCAAGAAGGGCAT
CAAAAGGCCGGCGGCCACGAAAAAGGCCGGCCAGGCAAAAAAGAAAAA
Gtag

Figure S2. Human codon-optimized BlatCas9 gene, Related to Figure 1.
Open reading frame of the BlatCas9 gene (highlighted in yellow), nuclear localization

signals (highlighted in blue) and stop codon (highlighted in red) are shown.

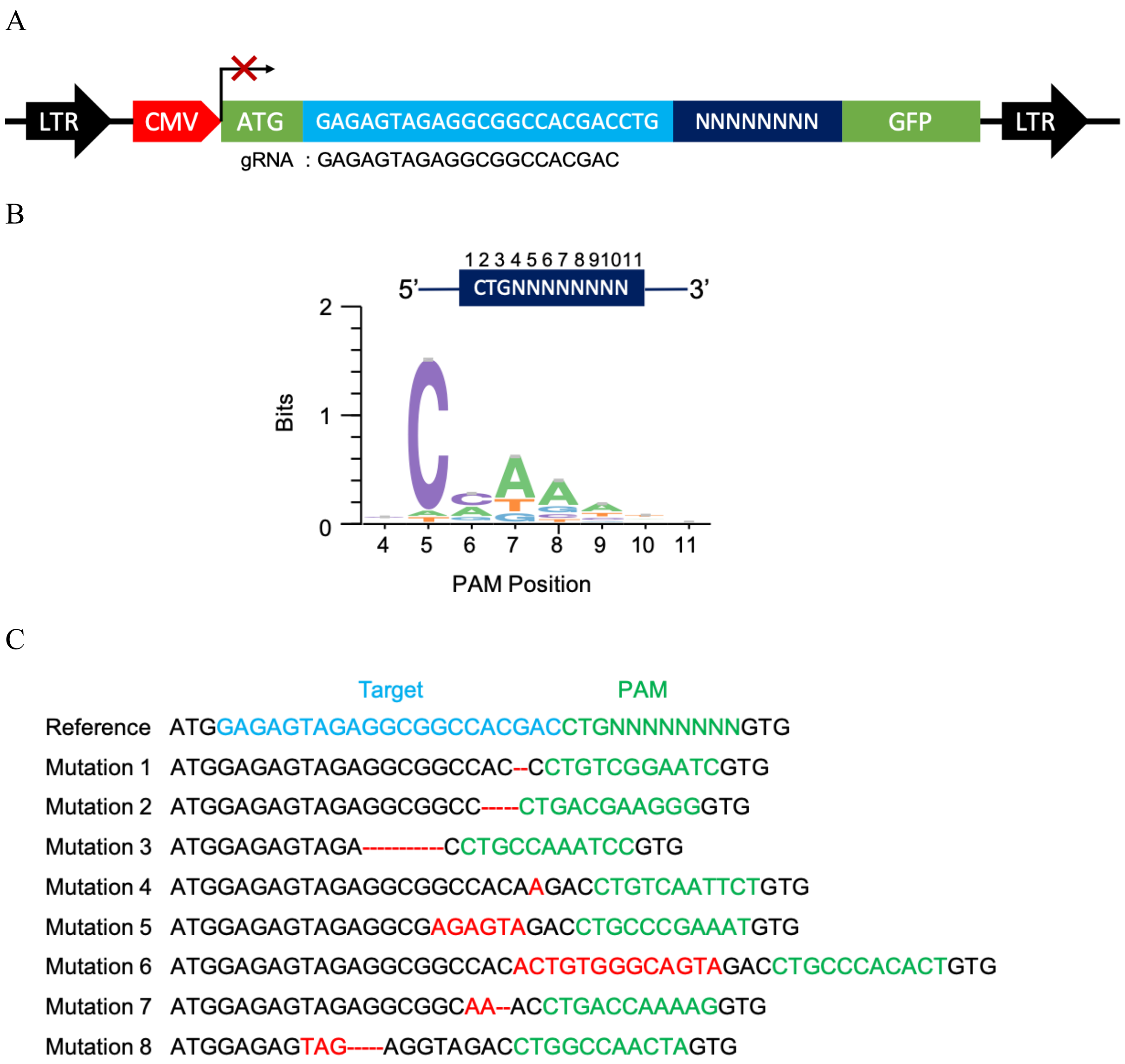

Figure S3. PAM sequence analysis for BlatCas9 on another target sequence, Related to Figure 2.

(A) Schematic diagram of the GFP reporter assay. The gRNA for PAM screening is shown below. (B) WebLogo is generated from deep sequencing data. (C) Deep sequencing shows indels occurred. Target sequence is shown in blue; indels are shown in red; CTG and 8-bp random sequences are show in green.

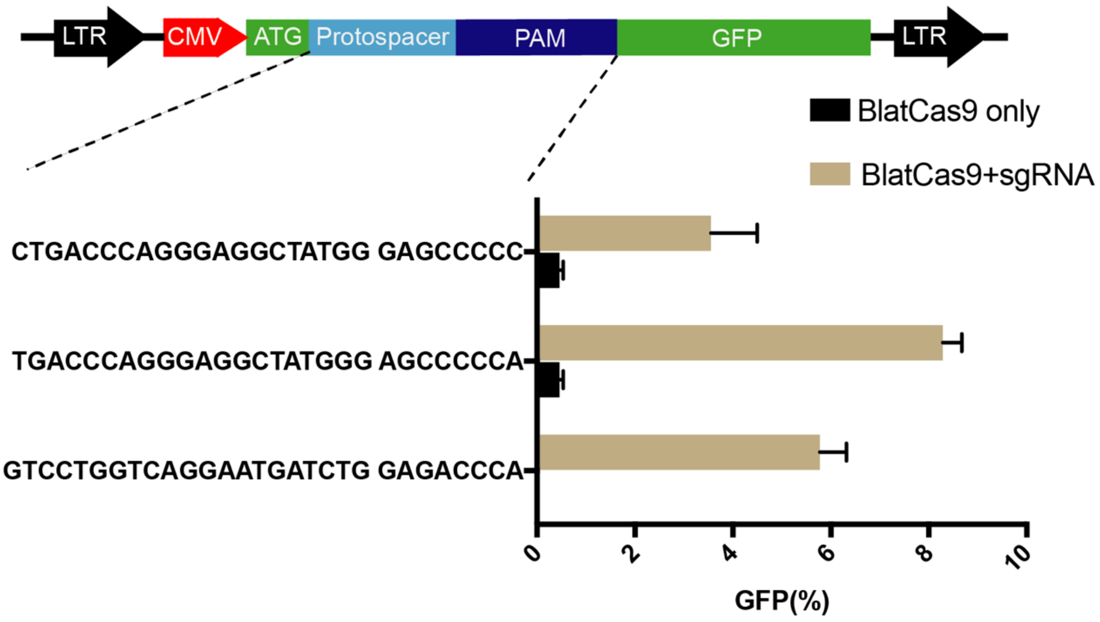

Figure S4. BlatCas9 can accept C at position 7 of PAM, Related to Figure 2. Three targets containing PAMs with C at position 7 are used to test whether BlatCas9 can accept C at position 7 of PAM.

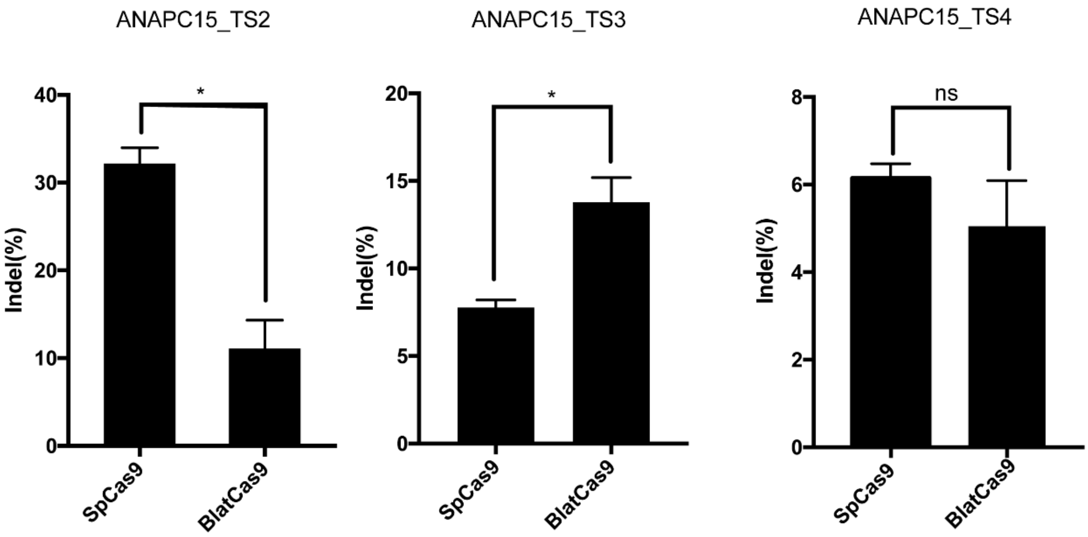

Figure S5. Comparison of SpCas9 and BlatCas9 efficiency, related to Figure 3. Three genomic loci containing PAMs which can be edited by both SpCas9 and BlatCas9 are selected.

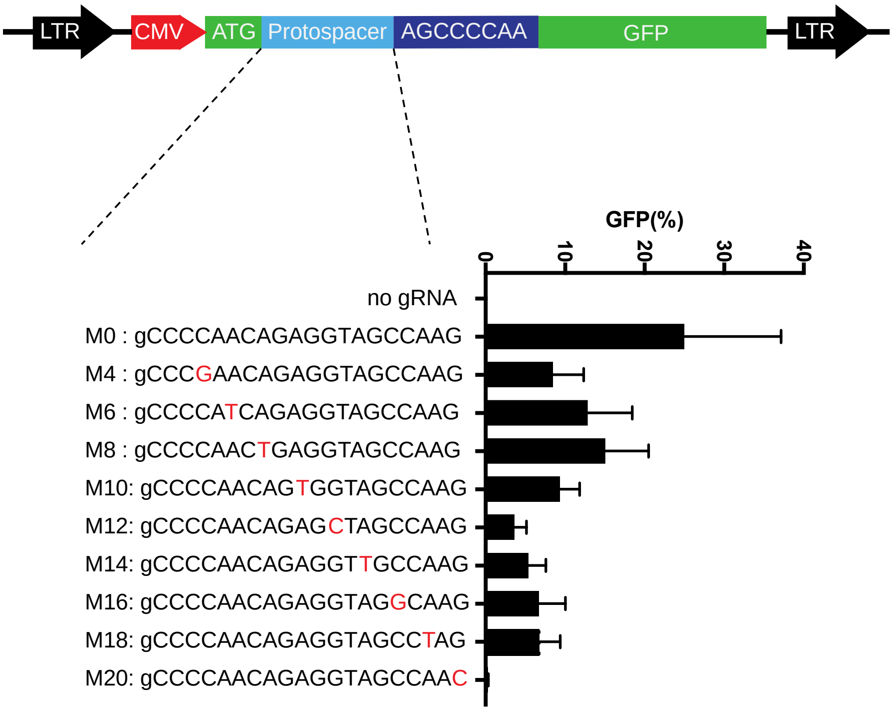

Figure S6. Analysis of BlatCas9 Specificity, Related to figure 6. A target sequence is inserted between ATG and GFP coding sequence, disrupting GFP expression. Target cleavage will induce GFP expression. A panel of gRNAs with single nucleotide mismatch (red) and each gRNA activity are shown below.
